# Reducing Dzyaloshinskii-Moriya interaction and field-free spin-orbit torque switching in synthetic antiferromagnets

**DOI:** 10.1038/s41467-021-23414-3

**Published:** 2021-05-25

**Authors:** Ruyi Chen, Qirui Cui, Liyang Liao, Yingmei Zhu, Ruiqi Zhang, Hua Bai, Yongjian Zhou, Guozhong Xing, Feng Pan, Hongxin Yang, Cheng Song

**Affiliations:** 1 Key Laboratory of Advanced Materials (MOE), School of Materials Science and Engineering, Beijing Innovation Center for Future Chip, Tsinghua University, Beijing, China; 2 Ningbo Institute of Materials Technology and Engineering, Chinese Academy of Sciences, Ningbo, China; 3 Faculty of Science and Engineering, University of Nottingham Ningbo China, Ningbo, China; 4 Key Laboratory of Microelectronic Devices and Integrated Technology, Institute of Microelectronics, Chinese Academy of Sciences, Beijing, China; 5 University of the Chinese Academy of Sciences, Beijing, P. R. China; 6 Center of Materials Science and Optoelectronics Engineering, University of Chinese Academy of Sciences, Beijing, China

**Keywords:** Surfaces, interfaces and thin films, Spintronics

## Abstract

Perpendicularly magnetized synthetic antiferromagnets (SAF), possessing low net magnetization and high thermal stability as well as easy reading and writing characteristics, have been intensively explored to replace the ferromagnetic free layers of magnetic tunnel junctions as the kernel of spintronic devices. So far, utilizing spin-orbit torque (SOT) to realize deterministic switching of perpendicular SAF have been reported while a large external magnetic field is typically needed to break the symmetry, making it impractical for applications. Here, combining theoretic analysis and experimental results, we report that the effective modulation of Dzyaloshinskii-Moriya interaction by the interfacial crystallinity between ferromagnets and adjacent heavy metals plays an important role in domain wall configurations. By adjusting the domain wall configuration between Bloch type and Néel type, we successfully demonstrate the field-free SOT-induced magnetization switching in [Co/Pd]/Ru/[Co/Pd] SAF devices constructed with a simple wedged structure. Our work provides a practical route for utilization of perpendicularly SAF in SOT devices and paves the way for magnetic memory devices with high density, low stray field, and low power consumption.

## Abstract (web summary)

Synthetic antiferromagnets (SAF), formed out of alternating layers of a ferromagnet with neutral spacer combine technologically appealing properties of both antiferromagnets and ferromagnets. Here, Chen et al demonstrate controlled switching of an SAF, without the need for an applied magnetic field.

## Introduction

The magnetic tunnel junctions (MTJs) are widely adopted in electronic devices such as nonvolatile memory, advanced magnetic read heads, and sensors because of their high magnetoresistance^1–3^. In conventional MTJ structures, the ferromagnetic free layer can be switched by spin-transfer torque via driving a current through a MTJ^4,5^. Nevertheless, ferromagnetic free layers in MTJs suffer from fundamental limits; in particular, the stray field interaction hinders the bit size, which is unfavorable for higher storage density and further device miniaturization. Owning to the strong anti-interference, ultrafast spin dynamics, and switching speed, the antiferromagnets exhibiting zero net magnetization and negligible stray field demonstrate a great potential in applications of reliable, high-speed, and high-density information storage^6–9^. However, researchers are encountering a dilemma in fact that the writing and reading of information in an antiferromagnet functional layer are not easy at the same time. Although several material systems have been proposed^10,11^, it is still difficult to experimentally manipulate and detect antiferromagnetic storage layers reliably.

SAF based on the Ruderman–Kittel–Kasuya–Yosida (RKKY) interaction^12,13^, where the top and bottom ferromagnets are antiferromagnetically coupled through a non-magnetic spacer layer with appropriate thickness, combines the advantages of zero stray field and high stability from antiferromagnets, as well as easy reading and writing characteristics in ferromagnets. In fact, utilizing SAF as the free layer in MTJ has been proposed with potential competitiveness in reducing the critical switching current and enhancing thermal stability^14,15^. Compared to conventional spin-transfer torque, spin–orbit torque is considered as an effective way to drive domain-wall motion and to switch magnetization with lower power consumption^16,17^. So far, SOT-induced magnetization switching has been widely investigated^17–26^ and extensive works have been done to eliminate the assistive magnetic field during SOT-induced magnetization switching with perpendicular magnetic anisotropy, including the design of interlayer exchange coupling^18^, spin–orbit effects with spin-rotation symmetry^20,21^, lateral structural asymmetry^22,23^, in-plane exchange bias^24,25^, and heavy metals with opposite spin Hall angle^26^. Several studies involving magnetization switching of SAF induced by SOT have been reported^27–30^; however, a relatively large in-plane magnetic field is needed to break the symmetry. Especially, the external assistive field should overcome the sum of the exchange coupling field and Dzyaloshinskii–Moriya interaction (DMI) effective field to achieve the deterministic switching^28^, making it impractical for applications. Therefore, field-free SOT-driven switching in SAFs remains to be demonstrated experimentally.

In this work, we systematically analyzed how to realize field-free magnetization switching in SAF by SOT and successfully demonstrated the deterministic switching experimentally. We show that the strength of DMI plays an important role in the configuration of domain walls, which further affects the current-induced magnetization switching. By means of interfacial engineering, the DMI between heavy metal and the ferromagnetic layer was greatly reduced, thereby reducing the assistive field required for SOT-induced magnetization switching. When the domain wall energy effective field is comparable with the DMI effective field, the domain walls exhibit the configuration between Bloch-type and Néel-type, making them easily manipulated by the external assistive magnetic field. In this case, to achieve the field-free SOT switching in SAF, a simple method of the wedged heavy metal structure is designed to break the symmetry, which enables the current-induced effective magnetic field. The SAF structure deposited on wedged Pt film presents uniform perpendicular magnetic anisotropy and the realization of field-free SOT switching in SAF shows stable circularity. We believe that these results represent an important step towards utilizing perpendicularly SAF as the free layer in MTJ devices and bringing functional SAF closer to potential applications.

## Results

### Model of SOT–induced magnetization switching in SAF

**Fig. 1:**
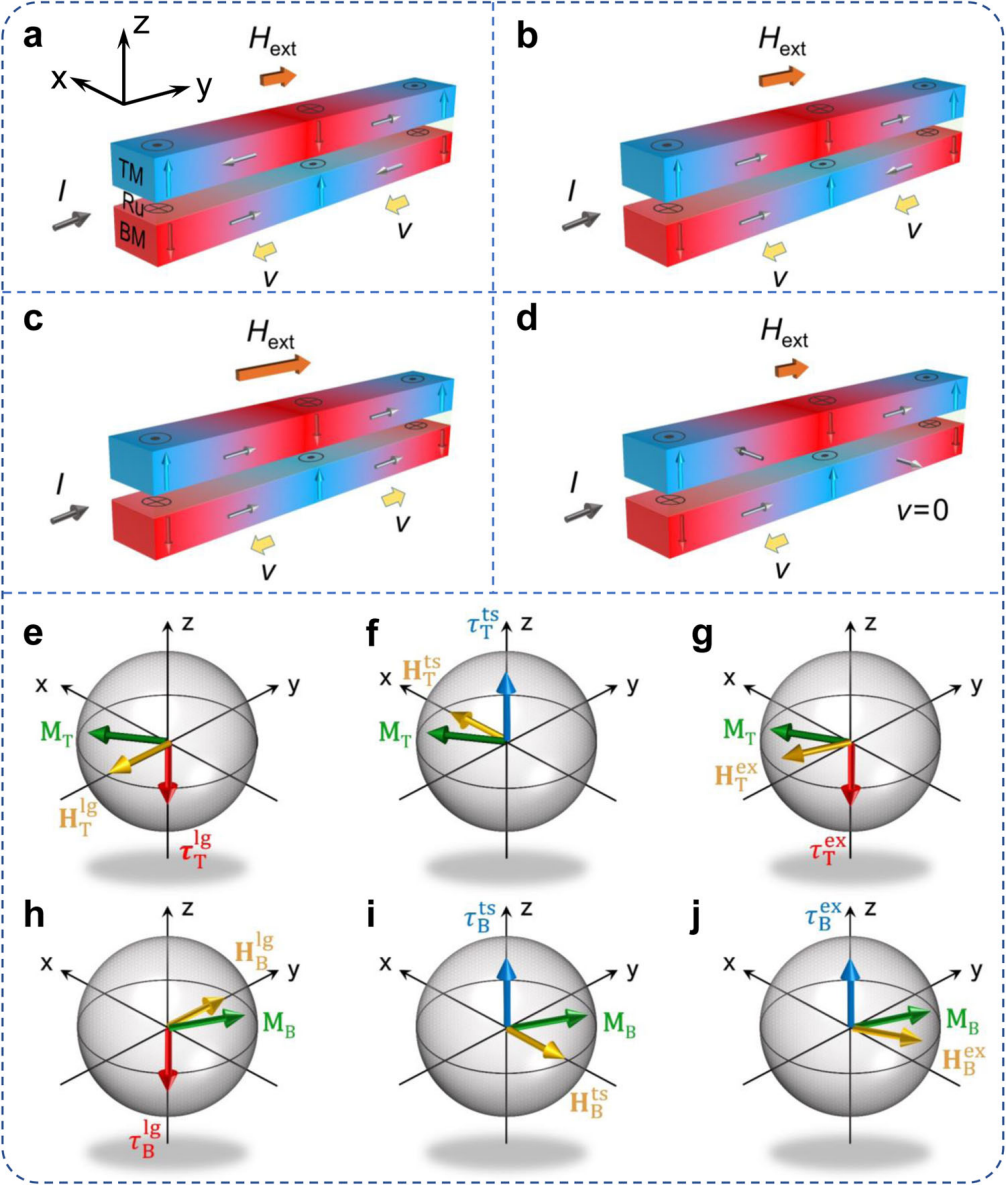
Schematic illustration of the domain wall configurations and torques in SAF. **a**–**d** Schematic illustrations of DWs in the top and bottom magnetic layers in perpendicularly magnetized SAF nanowires under an external magnetic field with *H*_DMI_ much larger than *H*_DWE_ (**a**–**c**) and *H*_DMI_ comparable to *H*_DWE_ (**d**). In the case of *H*_DMI_ much larger than *H*_DWE_, the center moments of domain walls are rotated to different directions with *H*_ext_ < *H*_EX_ (**a**), *H*_DMI_ < *H*_ext_ < *H*_DMI_ + *H*_EX_ (**b**), and *H*_DMI_ + *H*_EX_ < *H*_ext_ (**c**). Here, we assume the *H*_EX_ < *H*_DMI_. *H*_DWE_, *H*_DMI_ *H*_EX_, and *H*_ext_ are the domain wall energy effective field, interfacial DMI effective field, exchange coupling field, and external magnetic field, respectively, which give rise to ${H}_{{\rm{B}}}^{{\rm{lg}}}$, ${H}_{{\rm{T}}}^{{\rm{lg}}}$, ${H}_{{\rm{B}}}^{{\rm{ts}}}$, ${H}_{{\rm{T}}}^{{\rm{ts}}}$, ${H}_{{\rm{B}}}^{{\rm{ex}}}$, and ${H}_{{\rm{T}}}^{{\rm{ex}}}$. **e**–**j** Directions of different fields and corresponding longitudinal torques ${\tau }_{{\rm{B}}}^{{\rm{lg}}}$ and ${\tau }_{{\rm{T}}}^{{\rm{lg}}}$ (**e**, **h**), transversal torques ${\tau }_{{\rm{B}}}^{{\rm{ts}}}$ and ${\tau }_{{\rm{T}}}^{{\rm{ts}}}$ (**f**, **i**) and exchange torques ${\tau }_{{\rm{B}}}^{{\rm{ex}}}$ and ${\tau }_{{\rm{T}}}^{{\rm{ex}}}$ (**g**, **j**) shown schematically in the SAF, where *H*_DMI_ is comparable to *H*_DWE_. In each panel, upper and lower diagrams correspond to TM and BM, respectively.

We first describe our approach to realizing SOT switching without an external field in SAFs. In conventional SOT heterostructures with HM/FM layers, SOT can originate from the bulk spin Hall effect^31,32^ (SHE) and the interfacial Rashba effect^33,34^, which lead to two orthogonal components: the Slonczewski-like torque (damping-like torque) ***m*** $\times$ ***m*** $\times$ ***σ*** and the field-like torque ***m*** $\times$ ***σ***, where ***m*** is the unit vector of magnetization and ***σ*** is the spin polarization vector. The damping-like torque is originated mainly from the SHE and is responsible for the current-driven domain wall motion and magnetization switching. In the SAF structure where the top (TM) and bottom (BM) magnetic layer are coupled by a non-magnetic spacer, the magnetization of these two layers is antiparallelly aligned all the time as shown in Fig. 1a. The magnetization switching behavior can be explained from the perspective of SHE and DMI. Since the strong spin–orbit coupling and the resultant DMI at the interface of HM/BM, the BM domain wall “↓→↑” and “↑←↓” are Néel-type with left-hand chirality. When a current is injected into a heavy metal, the electrons with spin-polarization ***σ*** along the *y*-axis accumulate on the FM layer and generate an out-of-plane SOT effective field at the center of the domain wall. Considering the strength of DMI, the configuration of the domain wall can be divided into two types. It is noted that a Néel-type domain wall is preferred when the DMI effective field (*H*_DMI_) is much larger than the domain wall energy effective field (*H*_DWE_) whereas the domain wall exhibits the state between Néel-type and Bloch type when *H*_DMI_ is comparable to *H*_DWE_. In the former case, magnetization switching or domain wall motion can be realized by tuning the magnitude of the in-plane magnetic field. When the external field (*H*_ext_) is smaller than *H*_EX_, current-induced effective fields show opposite directions at two sides of the domain wall center, which lead to the domain wall movement in the same direction as illustrated in Fig. 1a. By gradually increasing the magnetic field to *H*_DMI_ < *H*_ext_ < *H*_DMI_ + *H*_EX_, the center magnetization of the domain walls in TM is rotated to the same directions whereas the magnetization alignments of BM still maintain the chirality and the deterministic switching cannot be realized as shown in Fig. 1b. Finally, in Fig. 1c, both the center moments of TM and BM domain walls align along the external magnetic field direction as this assistive field is large enough to overcome the sum of *H*_DMI_ and *H*_EX_. Due to the breaking of domain wall chirality, current generates the effective field in +z direction for both “↓→↑” and “↑→↓” domain walls, causing the expansion of the up domain under the SOT effective field. This is exactly the mechanism to realize SOT switching in SAF as reported before^28^.

However, the most striking problem is that a large external magnetic field is needed to break the symmetry, making it impractical for applications. We next ask whether a simple method can be proposed to reduce or eliminate the assistive magnetic field of SOT-induced SAF switching. As discussed above, the strength of DMI is an important factor that determines the domain wall configurations. We now focus on the case where *H*_DMI_ is comparable to *H*_DWE_. The competition between domain wall energy and DMI energy in this system leads to the alignment of domain wall moments between Néel-type and Bloch-type. As a result, the moments in the domain wall become sensitive to the external field and their alignment can be manipulated even a small in-plane magnetic field is applied as illustrated schematically in Fig. 1d. Consequently, the “↓→↑” domain wall can move under the applied current, however, the “↑☉↓” domain wall remains static until it is swallowed up by a domain wall further away. Finally, the whole BM domain is reversed by the current-induced SOT and the magnetization of TM is switched simultaneously through the antiferromagnetic coupling.

On the other hand, the spin Hall torque generated from the SOC layer results in the rotation of BM and TM moments from their equilibrium conditions which makes them subjected to several torques. Figure 1e–g presents the longitudinal field torque ${\tau }_{{\rm{T}}}^{{\rm{lg}}}$ which is derived mainly from the DMI effective field and external magnetic field, the DWE effective field-derived transverse field torque ${\tau }_{{\rm{T}}}^{{\rm{ts}}}$, and the antiferromagnetic exchange coupling field-induced exchange torques ${\tau }_{{\rm{T}}}^{{\rm{ex}}}$, respectively, acting on the top magnetic moments. Similarly, *M*_B_ is subjected to the corresponding effective fields (${H}_{{\rm{B}}}^{{\rm{lg}}}$, ${H}_{{\rm{B}}}^{{\rm{ts}}}$, ${H}_{{\rm{B}}}^{{\rm{ex}}}$) and torques (${\tau }_{{\rm{B}}}^{{\rm{lg}}}$, ${\tau }_{{\rm{B}}}^{{\rm{ts}}}$, ${\tau }_{{\rm{B}}}^{{\rm{ex}}}$) as illustrated in Fig. 1h–j. It is clearly shown that all these torques acting on the center moments of the domain wall are along the *z*-axis, which contributes to the motion of domain walls. In addition, the exchange torques that existed in SAF structures drive the TM and BM domain walls in the same direction, which is promising to promote the speed of domain wall motion, dramatically^35^. Considering that such faster SOT-assisted domain wall propagation would induce higher magnetization switching speed^36^, the switching in SAF is most likely faster than its ferromagnetic counterpart.

### Determination of Dzyaloshinskii–Moriya interaction effective field

**Fig. 2:**
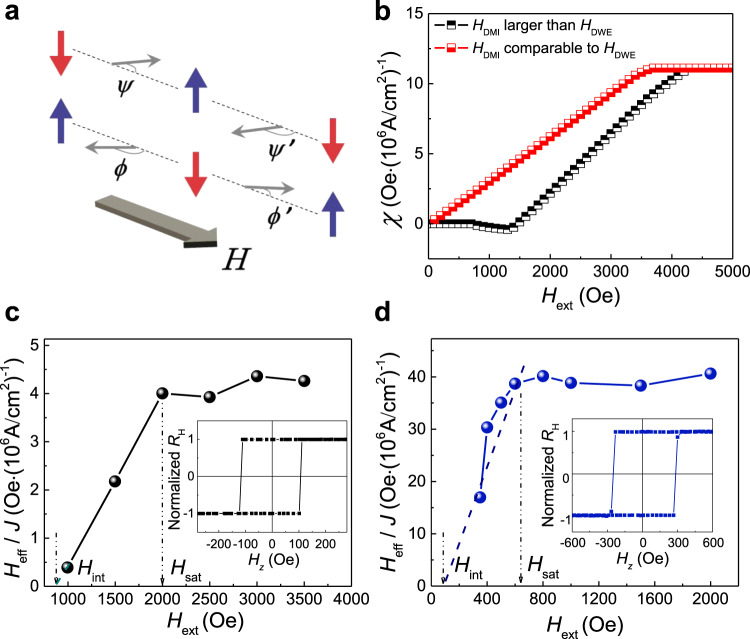
Determination of Dzyaloshinskii–Moriya interaction effective field and calculated SOT efficiency. **a** Sketch of the collective domain wall model. *Φ* and *Ψ* are the angles between the applied current and the up-to-down and down-to-up domain wall, while *Φ’* and *Ψ’* are corresponding angles with opposite directions. **b** Calculated results of SOT efficiency as a function of the external field for *H*_DMI_ much larger than *H*_DWE_ and *H*_DMI_ comparable to *H*_DWE_ in the SAF samples, respectively. **c**, **d** The SOT efficiency as a function of the external field for the ferromagnetic sample grown by e-beam evaporation (**c**) and magnetron sputtering (**d**), respectively. Insets are the corresponding normalized AHE curves for the ferromagnetic sample. *H*_sat_ is the external magnetic field at which the SOT efficiency gets saturated and *H*_int_ is the initial field to generate SOT efficiency. The dashed lines are the fitting lines of SOT efficiency from *H*_int_ to *H*_sat_.

To find out the origin of the *H*_DMI_ influence on the deterministic SOT switching, we derived the *H*_ext_ dependency of SOT efficiency *χ*. Figure 2a shows both the down-to-up domain walls and up-to-down domain walls in SAF structures where *Φ* and *Ψ* are the angles between the applied current and the up-to-down and down-to-up domain wall. Considering the collective domain wall model, the total domain wall energy of the up-to-down domain wall in SAF is expressed as^28,37,38^

1 $${\sigma }_{{\rm{DW}}}\left({H}_{{\rm{ext}}},\varPhi ,\varPsi \right)	= {\sigma }_{{\rm{B}}}+{\sigma }_{{\rm{T}}}+2{K}_{{\rm{D}}}\lambda \left({{{\cos }}}^{2}\varPhi +{{{\cos }}}^{2}\varPsi \right)\\ 	\quad-\pi \lambda {M}_{{\rm{B}}}\left({H}_{{\rm{ext}}}+{H}_{{\rm{DMI}}}^{{\rm{B}}}\right){{\cos }}\varPhi -\pi \lambda {M}_{{\rm{T}}}\\ 	\quad\times\left({H}_{{\rm{ext}}}-{H}_{{\rm{DMI}}}^{{\rm{T}}}\right){{\cos }}\varPsi +\pi \lambda {J}_{{\rm{EX}}}{{\cos }}\left(\varPhi -\varPsi \right)$$

where ${\sigma }_{{\rm{B}}}$ and ${\sigma }_{{\rm{T}}}$ are the Bloch-type domain wall energy densities of the bottom and upper domain wall, respectively, *K*_D_ is the domain wall anisotropy energy density, *λ* is the domain wall width, *J*_EX_ is the interlayer coupling strength, ${H}_{{\rm{DMI}}}^{{\rm{B}}}$ and ${H}_{{\rm{DMI}}}^{{\rm{T}}}$are the DMI effective fields of the BM and TM, respectively. By solving the equation at two typical conditions of *H*_DMI_ (Supplementary Note 1), Fig. 2b depicts the SOT efficiency as a function of the external field for *H*_DMI_ much larger than *H*_DWE_ and *H*_DMI_ comparable to *H*_DWE_ in the SAF samples, respectively. For *H*_DMI_ much larger than *H*_DWE_, an external assistive field is needed to achieve the SOT switching; however, the current-induced SOT switching becomes easier when the *H*_DMI_ is comparable to *H*_DWE_, which is consistent with the conclusions discussed earlier.

We then focus on the strength of interfacial DMI between the heavy metal and bottom magnetic layer. Stacks of Ta(2)/Pt(4)/[Co(0.46)/Pd(0.8)]_2_/Co(0.46)/Pd(2) and Ta(2)/Pt(4)/Co(0.3)/Pd(0.5)/Co(0.3)/Pd(2) (units in nanometer) were deposited via dc magnetron sputtering and e-beam evaporation, respectively. To determine the interfacial DMI effective field in this system, we measured the current-induced SOT efficiency in the ferromagnetic layer. When an in-plane assistive field is applied, the out-of-plane equivalent field induced by SOT can be reflected in the switching efficiency^28,39,40^. In Fig. 2c, d, we present the SOT efficiency as a function of the external field for the perpendicular ferromagnetic sample. Inserts are the anomalous Hall effect (AHE) curves of the ferromagnetic stack films. The typical sharp square shape of the AHE curves of these two samples indicates the perpendicular magnetic anisotropy characteristic. The current density *J* is calculated assuming a uniform current distribution. *H*_eff_ is obtained by recording the Hall resistance by rotating the fixed magnitude external field around the film plane. It is noted that the SOT-induced deterministic switching does not occur until a moderate magnitude of *H*_ext_ is applied. For the sample grown by e-beam evaporation, 1  kOe of the external field is needed to observe the SOT equivalent field while 350  Oe is enough for the sputtering sample. Another striking feature is that the efficiency almost gets saturated when the external field is up to 2  kOe (e-beam evaporation) and 620  Oe (magnetron sputtering) for the two systems. Thus, a *H*_DMI_ of about 1450  Oe and 360  Oe, respectively, is obtained by calculating (*H*_sat_ + *H*_int_)/2 and the *H*_DWE_ is subsequently determined as 550  Oe for the evaporation sample and 260  Oe for the sputtering sample. *H*_sat_ is the external magnetic field at which the SOT efficiency gets saturated and *H*_int_ is the initial field to generate SOT efficiency. Interestingly, the measured *H*_DMI_ in the sample grown by sputtering is much smaller than the sample grown via e-beam evaporation and the reason will be discussed in detail in the following.

### SAF structures grown on the top of the wedged films

**Fig. 3:**
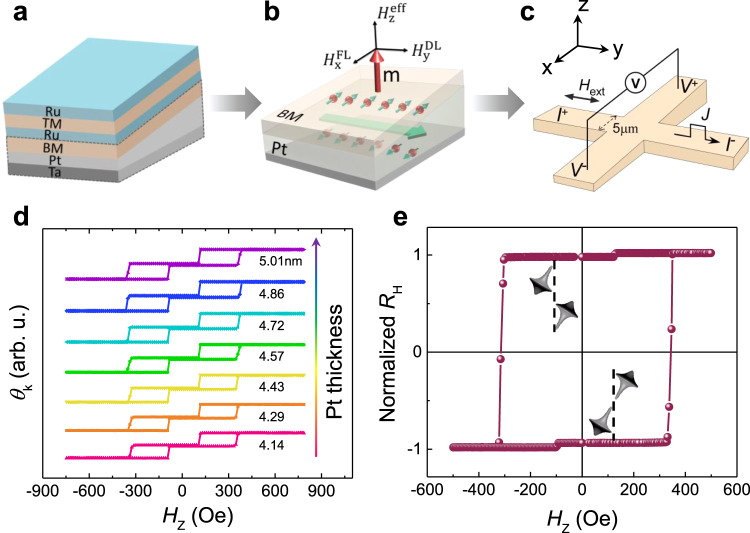
Current-induced out-of-plane effective magnetic field (${H}_{{\rm{z}}}^{{\rm{eff}}}$) by depositing a wedged asymmetric layer of Pt. **a** Schematic of the studied Ta/Pt/[Co/Pd]_2_/Co/Ru/[Co/Pd]_3_/Co/Ru multilayer. The wedged Pt layer was grown through a moving baffle. **b** Creation of a net out-of-plane spin polarization and effective magnetic field in the studied SAF structure. ${H}_{{\rm{y}}}^{{\rm{DL}}}$ and ${H}_{{\rm{x}}}^{{\rm{FL}}}$ are the damping-like and field-like magnetic fields, respectively. ${H}_{{\rm{z}}}^{{\rm{eff}}}$ is the effective perpendicular magnetic field and ***m*** represents the unit vector of the magnetization. The wedged Pt layer allows for the generation of ${H}_{{\rm{z}}}^{{\rm{eff}}}$. **c** Schematic of the Hall bar and the measurement configuration. *I* stands for current and *V* parameters voltage, and *H*_ext_ is external field. **d** Out-of*-*plane field (*H*_z_) dependent Kerr signals (*θ*_k_) of the compensated SAF sample at different nominal Pt thicknesses. The antiferromagnetic coupling strength of the sample hardly depends on the thickness of the wedged layer. **e** *R*_H_ curves of SAF sample measured when sweeping an external field along the *z*-direction. The inset represents the spin configuration of the two antiparallel states.

Based on the discussion above, the SOT-induced magnetization switching in SAF becomes easier since we have confirmed the *H*_DMI_ in our sample grown by sputtering is comparable to the *H*_DWE_. Here, a SAF stack of Ta(2)/Pt(wedged)/BM/Ru/(0.68)/TM/Ru(2) is deposited via dc magnetron sputtering where the BM is [Co(0.46)/Pd(0.8)]_2_/Co(0.46) and TM is Co(0.46)/Pd(0.8)]_3_/Co(0.46). To achieve the antiferromagnetic coupling, the thickness of the spacer Ru is chosen as 0.68 nm. The sample layout is displayed in Fig. 3a and the wedged Pt layer was grown through a moving baffle during the deposition. As shown in Fig. 3b, when the current is applied, two orthogonal effective fields, namely ${H}_{{\rm{y}}}^{{\rm{DL}}}$ and ${H}_{{\rm{x}}}^{{\rm{FL}}}$ corresponding to damping-like torque and field-like torque, are created by SHE and the interfacial Rashba effect. Besides, due to the wedged spin–orbit coupling (SOC) layer, the mirror symmetry of the *yz-*plane is broken, which allows for the creation of an out-of-plane effective field ${H}_{{\rm{z}}}^{{\rm{eff}}}$ whose direction depends on the current polarity^22,23,41^. To check the quality of the SAF sample grown on wedged Pt layer, the as-deposited films were studied using the magneto-optical Kerr effect (MOKE). It is clearly shown in Fig. 3d that the BM and TM are antiferromagnetically coupled where two switching steps are observed, representing the separate magnetization switching of top and bottom ferromagnetic layer, respectively. The negligible net magnetization at zero magnetic fields indicates the antiparallel alignment of the two magnetic layers which is immune to external magnetic fields. Note that the coercivity and perpendicular magnetic anisotropy at different thickness of Pt shows almost no difference, suggesting the uniformity of SAF grown on wedged structures. Then, the multilayers were patterned into Hall bar devices with a channel width of 5 μm by utilizing photolithography and Ar ion etching (Fig. 3c). Figure 3e presents the anomalous Hall effect curves in which the two completely compensated states are separated in electrical measurements. This can be understood from that the anomalous Hall coefficients of BM and TM layer are different, which can be ascribed to the sample growth condition. The anomalous Hall coefficient of [Co/Pd] multilayers grown on Ru is usually smaller than that deposited on Pt because of the smaller spin–orbit coupling of Ru^28,42,43^.

### Current-induced spin–orbit torque switching in SAF

**Fig. 4:**
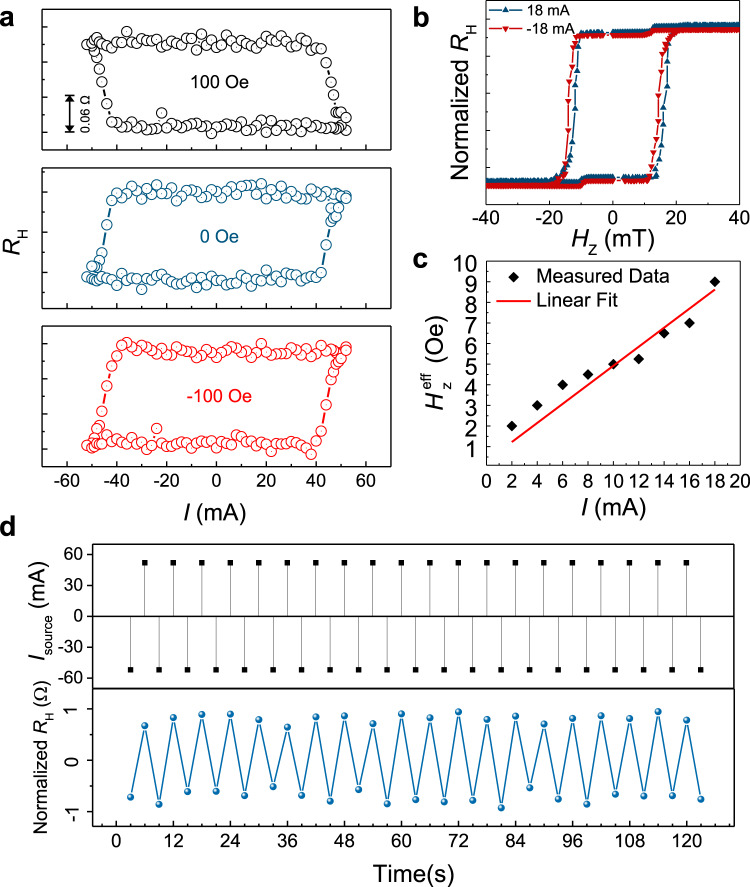
Field-free spin–orbit torque switching in SAF. **a** SOT switching under different external assistive magnetic fields. **b** Anomalous Hall effect measured at opposite current polarities. Here, *R*_H_ is the normalized anomalous Hall resistance and *H*_Z_ is the applied out-of-plane magnetic field. The Pt thickness in this device is 4.43 nm. **c** Applied current dependent current-induced perpendicular effective magnetic field, exhibiting a linear relationship. **d** zero field repeatable SOT switching under successive current pulses with opposite polarities. The duration of the current pulse is 1 ms and the period is 3 s.

We now turn to the current-induced magnetization switching by means of SOT in SAF structures. For these measurements, the Hall resistance was recorded while sweeping the current pulses along the *y*-direction and the external magnetic fields were applied along the current direction. The most eminent feature in Fig. 4a is that the deterministic magnetization switching induced by the current was realized without any external fields. The switching is clockwise for positive *H*_ext_ (+100 Oe) and anticlockwise for negative *H*_ext_ and zero field (–100 Oe and 0 Oe). Especially, for the field-free case, a large positive current of 50 mA leads to the “↓↑” alignment of magnetization and the low resistance state “↑↓” is preferred upon applying the corresponding negative current. It is worth noting that the switching was not observed when the current is applied parallel to the gradient direction of the wedged films (Supplementary Fig. 1), similar to the asymmetric structure induced field-free SOT switching in ferromagnets. To further figure out the current-induced effective magnetic field, AHE was measured with the current applied perpendicular to the gradient direction of the wedged films. As depicted in Fig. 4b, hysteresis loops under opposite current polarities of +18 mA and –18 mA are shifted to two positive and negative directions, respectively, indicating the current-induced opposite directions of ${H}_{{\rm{z}}}^{{\rm{eff}}}$ in SAF structure. Furthermore, a linear relationship between the current-induced out-of-plane effective magnetic field and the applied current was observed in Fig. 4c. The ${H}_{{\rm{z}}}^{{\rm{eff}}}$ is obtained by extracting the offset from zero field in the AHE curves. What is more, the repeatability of this field-free SOT switching was investigated by applying successive positive and negative current pulses to the device. Figure 4d presents the successive SOT switching at zero field with 1 ms duration of current pulses and a period of 3 s. Obviously, the high resistance state and low resistance state, which correspond to the two antiparallel alignments of moments for TM and BM, can be switched cyclically under a series of current pulses. To further confirm the transport signals above arise from the SOT-induced magnetization switching rather than some electric signals due to non-magnetic origin^44^, we used MOKE microscopy to check the magnetization states of an uncompensated SAF sample (Supplementary Note 2) before and after SOT switching. The cycling contrast change of the Hall channel after applying positive and negative current pulses further confirms the fully SOT switching in the SAF structure. Therefore, we conclude that the SAF structures grown on a wedged SOC layer show uniform anisotropy and exhibit quite stable current-induced magnetization switching performance in the absence of an external field, bringing SAF closer to potential applications.

### Interfacial characterization and first-principles calculations

**Fig. 5:**
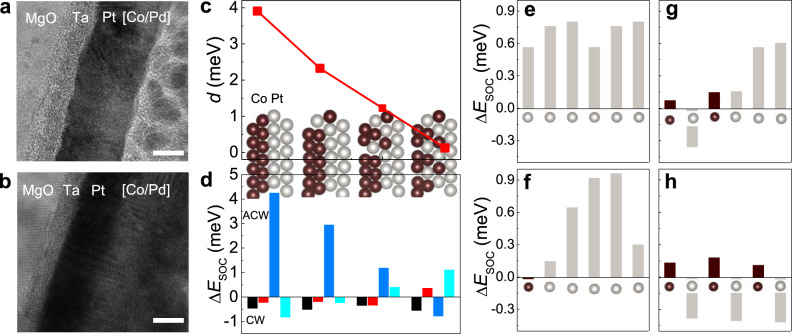
Cross-sectional TEM images and energy source of DMI from first-principles calculations. **a**, **b** High-resolution TEM images of the SAF and ferromagnetic structures were grown by magnetron sputtering (**a**) and e-beam evaporation (**b**), respectively. The scale bar in TEM images corresponds to 5 nm. **c**, **d** Total DMI coefficient *d* (**c**), and layer-resolved SOC energy difference $\triangle {E}_{{\rm{soc}}}$ (**d**) of opposite chirality of Pt/Co heterostructures with increasing interfacial roughness. **e**–**h** Atomic-resolved $\triangle {E}_{{\rm{soc}}}$ for each atom in interfacial Pt layer when intermixed with Co atoms.

To further comprehend the mechanism responsible for DMI engineering, the microstructure characterization and the first-principles calculations are carried out. Stacks of Ta/Pt/[Co/Pd]_3_ and Ta/Pt/[Co/Pd]_2_/Co/Ru/[Co/Pd]_3_/Co/Ru are grown by e-beam evaporation and magnetron sputtering as presented in Fig. 5a and b, respectively. The most striking feature is that the Pt and [Co/Pd] multilayers deposited by e-beam evaporation show consistent lattice fringes which exhibit better crystallinity than that grown by magnetron sputtering. Consequently, the alignment of the atoms at the Pt and Co interface differs dramatically between these two methods, leading to the variation of DMI whose strength is significantly affected by the interface conditions^45,46^. To further investigate the influence of interfacial roughness on DMI of Co/Pt structure, we constructed a series of heterostructures with different interfacial crystallinity by mixing the interfacial atoms as shown in the inset of Fig. 5c. The DMI strength $d$ is extracted by comparing the energy difference between clockwise (CW) and anticlockwise (ACW) chiral spin configurations in the 6 × 1 supercell (Supplementary Fig. 3)with the corresponding formula: $d=({E}_{{\rm{CW}}}-{E}_{{\rm{ACW}}})/9\sqrt{3}$. With the increasing of the interfacial Co–Pt intermixing, $d$ keeps decreasing (see red line in Fig. 5c), which is consistent with the experimental observations that better crystallinity results in larger DMI. To understand the mechanism for the variation of interfacial DMI, we calculated the layer-resolved SOC energy difference $\triangle {E}_{{\rm{soc}}}$ between the opposite chiral spin textures. As shown in Fig. 5d, the dominant contribution to the DMI stems from the adjacent Pt layers when the interface crystallinity is perfect, which is consistent with the Fert–Levy model^45^. Polarized electrons transfer between Co atoms through the mediate Pt atom, and the spin orientations of these electrons are scattered by the large spin–orbit coupling of Pt. When interfacial Pt is mixed with Co atoms, the Co–Co–Pt triplet is broken, which results in the contribution from Pt layer to total DMI of CW chirality decreased, and $\triangle {E}_{{\rm{soc}}}$ from different layer starts to cancel each other. We also calculate $\triangle {E}_{{\rm{soc}}}$ of each atom in the interfacial Pt layer as shown in Fig. 5e–h. As the interfacial crystallinity decreases, a contribution from each Pt atom to the DMI of CW chirality decreases and contributions to DMI from Pt and mixed Co atoms begin to cancel each other, which leads to the reduction of DMI of the interfacial Pt layer in Fig. 1d.

## Discussion

To sum up, we have systematically demonstrated the deterministic field-free magnetization switching in the perpendicular synthetic antiferromagnetic structures, in which the tailored strength of Dzyaloshinskii–Moriya interaction plays the major role in configuring domain walls with enhanced spin–orbit torque efficiency. When the domain wall energy effective field is comparable with DMI effective field, the domain wall exhibits the configuration between Bloch type and Néel type which can be manipulated by external field easily. By depositing the [Co/Pd]/Ru/[Co/Pd]/Ru structure on the wedged SOC layer, we obtained the completely compensated SAF with uniform anisotropy and finally realized the SOT-induced magnetization switching without any external fields, which is crucial for practical applications. We have also shown that the DMI strength depends dramatically on the crystallinity of the Pt/Co interface and the intermixing interface favors weaker DMI, which is beneficial for the field-free SOT switching. Our work provides a practical route for utilization of perpendicularly SAF in SOT devices and paves the way for magnetic memory devices with high density, low stray field, and low power consumption.

## Methods

### Sample preparation

The wedged Ta/Pt/[Co/Pd]_2_/Co/Ru/[Co/Pd]_3_/Co/Ru SAF films were deposited at room temperature onto 5 mm × 5 mm MgO substrate for magnetic property measurements via d.c. magnetron sputtering with a base vacuum better than 8.0 × 10^−5^ mTorr, and the working argon pressure was 3 mTorr. The wedged Pt layer was grown through a moving baffle during the deposition. The Ta/Pt/[Co/Pd]_2_/Co/Pd ferromagnetic stacks were deposited on a 5 mm × 5 mm MgO substrate via e-beam evaporation at a base pressure of 5 × 10^−6^  mTorr. Devices were patterned by means of standard photolithography and subsequent Ar ion milling.

### Magnetization and transport measurements

The anomalous Hall effect and current-induced magnetization switching were carried out by four-point measurements in a Hall cross with a channel width of 5 μm at room temperature. Magnetic domain images and hysteresis loops were captured using a MagVision Kerr Imaging System, which operates on the magneto-optical Kerr effect in the polar configuration. In the polar configuration, the out-of-plane magnetization is probed and observed as different levels of brightness in the image.

### First-principles calculations

Our first-principles calculations were carried out within the framework of density functional theory (DFT) as implemented in Vienna ab initio simulation package (VASP)^47–49^. The exchange and correlation functional are treated with the generalized gradient approximation (GGA) of Perdew–Burke–Ernzerhof (PBE) functional^50,51^. The energy cutoff for plane wave expansion is set to 350 eV, and a Г-centered k-point mesh of 3 × 18 × 1 is adopted in our calculations of Dzyaloshinskii–Moriya interaction (DMI) for the 6 × 1 supercell as shown in Supplementary Fig. 3. A vacuum space larger than 15 Å is applied to avoid interaction between two adjacent slabs. The geometry optimization is carried out until the Hellmann-Feynman force is less than 0.01 eV, and the convergence precision of total energy is set to 10^−7^  eV. The strength of DMI is calculated by using the constrained spin-spiral supercell method^45^.

## Supplementary information

Supplementary Information

## Data Availability

The data that support the findings of this study are available from the corresponding authors upon reasonable request.
