# Supplementary material for: Reducing Dzyaloshinskii-Moriya interaction and field-free spin-orbit torque switching in synthetic antiferromagnets
Source: Nat Commun. 2021 May 25;12:3113. doi: 10.1038/s41467-021-23414-3 (PMC8149869; doi:10.1038/s41467-021-23414-3)
Supplement: Supplementary file 1 — Supplementary Information [file 41467_2021_23414_MOESM1_ESM.pdf]

Supplementary Information

Reducing Dzyaloshinskii-Moriya interaction and field-free spin-orbit torque switching in synthetic antiferromagnets

Ruyi Chen,[1] Qirui Cui,[2,3] Liyang Liao,[1] Yingmei Zhu,[2] Ruiqi Zhang,[1] Hua Bai,[1] Yongjian Zhou,[1] Guozhong Xing,[4,5] Feng Pan,[1] Hongxin Yang,[2,6,*] Cheng Song[1,*]

[1]*Key Laboratory of Advanced Materials (MOE), School of Materials Science and Engineering, Beijing Innovation Center for Future Chip, Tsinghua University, Beijing 100084, China*

[2]*Ningbo Institute of Materials Technology and Engineering, Chinese Academy of Sciences, Ningbo 315201, China*

[3]*Faculty of Science and Engineering, University of Nottingham Ningbo China, Ningbo, China*

[4]*Key Laboratory of Microelectronic Devices and Integrated Technology, Institute of Microelectronics, Chinese Academy of Sciences, Beijing 100029, China*

[5]*University of the Chinese Academy of Sciences, Beijing, 100049, P. R. China*

[6]*Center of Materials Science and Optoelectronics Engineering, University of Chinese Academy of Sciences, Beijing 100049, China*

[*] E-mail: hongxin.yang@nimte.ac.cn;
songcheng@mail.tsinghua.edu.cn

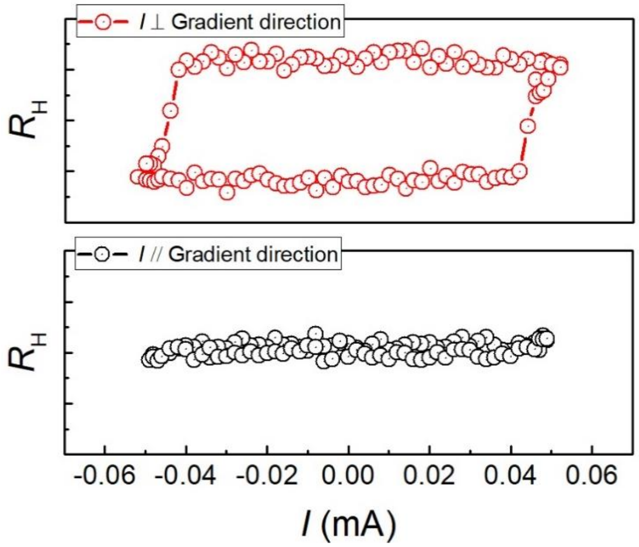

Supplementary Figure 1 | Spin-orbit torque switching in synthetic antiferromagnet structure without in-plane magnetic fields under the current direction perpendicular (top) and parallel (bottom) to the gradient direction of the wedged film. Obviously, the field-free SOT switching is achieved for the input current direction perpendicular to the gradient direction of the wedged film whereas no switching trend is observed when the current direction parallel to the gradient direction of the wedged film.

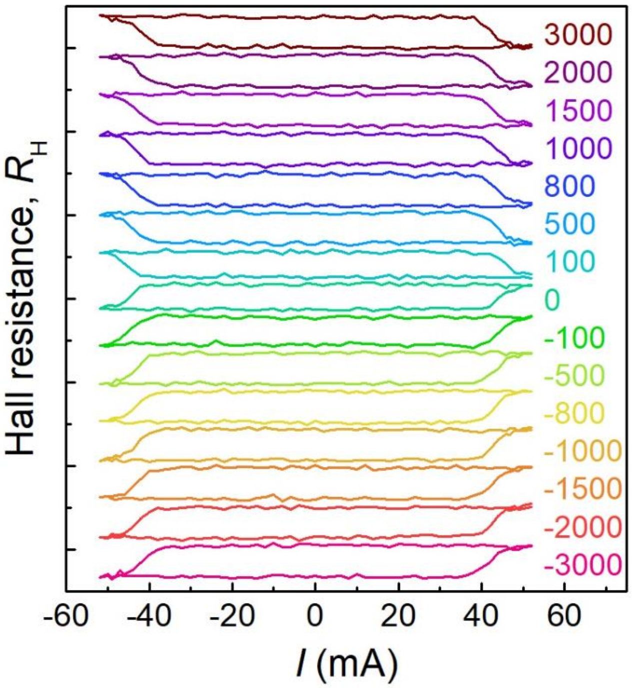

Supplementary Figure 2 | Current induced deterministic magnetization switching in SAF structure with different in-plane assistant magnetic fields. It is clear that the SOT polarity is reversed when changing the applied magnetic field from 3000 Oe to –3000 Oe. For the field free case, a large positive current of 50 mA leads to the "↓↑" alignment of magnetization and the low resistance state "↑↓" is preferred upon applying the corresponding negative current, exhibiting the same SOT switching polarity with the negative field case.

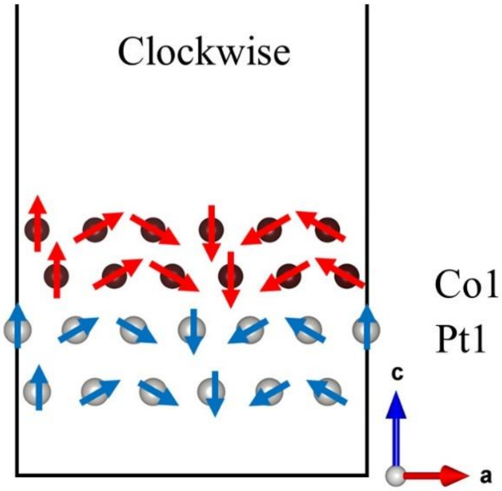

Supplementary Figure 3 | Examples of clockwise (left panel) and anticlockwise (right panel) spin configurations used to calculate the DMI of Co/Pt heterostructures.

Supplementary Note 1 | Collective domain wall model of synthetic antiferromagnets

For a conventional ferromagnetic structure, based on the multidomain SOT switching mechanism, SOT efficiency χ = H_{eff} /J_{C} (where J_{C} represents the current density in the heavy metal layer) is given by

$$\chi = \frac{\pi}{2}\frac{\hbar\theta_{\mathrm{SH}}}{2e\mu_0 M_{\mathrm{S}} t_{\mathrm{F}}}\cos\Phi \tag{S1}$$

where ħ, e and μ_0 are the reduced Planck constant, the electric charge and the permeability of vacuum, respectively, M_{S} is the saturated magnetization, t_{F} parameterizes the effective thickness of the ferromagnetic layer, θ_{SH} is the effective spin Hall angle, Φ is the angle between the central moment of the domain wall and the current, and $\cos\Phi$ is equal to 1 as the SOT efficiency is saturated. Here, in our system, we define the bottom magnetic layer as the effective SOT switching layer since the spin torque acts on the bottom magnetic layer and further leads to the top magnetic layer switching through the antiferromagnetic coupling. Consequently, we get the z-direction effective field of spin-orbit torque in the SAF structure approximately as

$$\chi = \frac{\pi}{2}\frac{\hbar\theta_{\mathrm{SH}}}{2e\mu_0 M_{\mathrm{B}} t_{\mathrm{B}}}\frac{(\cos\Phi+\cos\Phi')}{2} \tag{S2}$$

where M_{B} and t_{B} are the magnetization and effective thickness of bottom magnetic layer in SAF, respectively.

To find out the origin of the H_{DMI} influence on the deterministic SOT switching, we derived the H_{ext} dependency of SOT efficiency χ. Fig. 2(a) shows both the down-to-up domain walls and up-to-down domain walls in SAF structures where Φ and Ψ are the angles between the applied current and the up-to-down and down-to-up domain wall, while Φ' and Ψ' are corresponding angles with opposite directions. Considering the

collective domain wall model, the total domain wall energy of the up-to-down and down to up domain wall in SAF is expressed as

$$\sigma_{\mathrm{DW}}(H_{\mathrm{ext}}, \Phi, \Psi) = \sigma_{\mathrm{B}} + \sigma_{\mathrm{T}} + 2K_{\mathrm{D}}\lambda(\cos^2\Phi + \cos^2\Psi) - \pi\lambda M_{\mathrm{B}}\left(H_{\mathrm{ext}} + H_{\mathrm{DMI}}^{\mathrm{B}}\right)\cos\Phi - \pi\lambda M_{\mathrm{T}}\left(H_{\mathrm{ext}} - H_{\mathrm{DMI}}^{\mathrm{T}}\right)\cos\Psi + \pi\lambda J_{\mathrm{EX}}\cos(\Phi - \Psi), \quad \text{(S3)}$$

$$\sigma_{\mathrm{DW}}(H_{\mathrm{ext}}, \Phi', \Psi') = \sigma_{\mathrm{B}} + \sigma_{\mathrm{T}} + 2K_{\mathrm{D}}\lambda(\cos^2\Phi' + \cos^2\Psi') - \pi\lambda M_{\mathrm{B}}\left(H_{\mathrm{ext}} - H_{\mathrm{DMI}}^{\mathrm{B}}\right)\cos\Phi' - \pi\lambda M_{\mathrm{T}}\left(H_{\mathrm{ext}} + H_{\mathrm{DMI}}^{\mathrm{T}}\right)\cos\Psi' + \pi\lambda J_{\mathrm{EX}}\cos(\Phi' - \Psi') \quad \text{(S4)}$$

respectively, where σ_{B} and σ_{T} are the Bloch-type domain wall energy densities of the bottom and upper domain wall, respectively, K_{D} is the domain wall anisotropy energy density, λ is the domain wall width, J_{EX} is the interlayer coupling strength, $H_{\mathrm{DMI}}^{\mathrm{B}}$ and $H_{\mathrm{DMI}}^{\mathrm{T}}$ are the DMI effective fields of the BM and TM, respectively. In order to find the influence of the interfacial DMI on SOT, the external magnetic field dependence of spin orbit efficiency at two typical conditions are studied. For the H_{DMI} much larger than H_{DWE}, parameters are set as M_{B} =1450 emu/cm^3, M_{T} = 1400 emu/cm^3, $H_{\mathrm{DMI}}^{\mathrm{B}}$ = 1500 Oe, $H_{\mathrm{DMI}}^{\mathrm{T}}$ = 800 Oe, $K_{\mathrm{B}} = 3.2\times10^6$ erg/cm^3, $K_{\mathrm{T}} = 2.8\times10^6$ erg/cm^3, $J_{\mathrm{EX}} = 2\times10^6$ erg/cm^3, $4K_{\mathrm{D}}/\pi M_{\mathrm{B}}$ = 400 Oe. On the other hand, for the H_{DMI} comparable to H_{DWE}, parameters are set as M_{B} =1450 emu/cm^3, M_{T} = 1400 emu/cm^3, H_{DMI} = 360 Oe, $H_{\mathrm{DMI}}^{\mathrm{T}}$ = 100 Oe, $K_{\mathrm{B}} = 3.2\times10^6$ erg/cm^3, $K_{\mathrm{T}} = 2.8\times10^6$ erg/cm^3, $J_{\mathrm{EX}} = 2\times10^6$ erg/cm^3, $4K_{\mathrm{D}}/\pi M_{\mathrm{B}}$ = 400 Oe. By appling these parameters to Eq. (S3) and (S4), we could get the Φ, Ψ, Φ', and Ψ' for each given H_{ext} via mimimizing the domain wall energy σ_{DW}.

Supplementary Note 2 | MOKE imaging of the uncompensated SAF specimen during field-free SOT switching.

To further confirm the SOT-induced magnetization switching, a SAF stack of Ta(2)/Pt(wedged)/BM/Ru/(0.68)/TM/Ru(2) (units in nanometers) was deposited via dc magnetron sputtering where both BM and TM are $[Co(0.46)/Pd(0.8)]_3/Co(0.46)$ multilayers. Supplementary Figure 4 displays the out-of-plane field dependent Kerr signals of the uncompensated SAF sample, where the top and bottom magnetic layers are not completely compensated at zero field due to different magnetic dead layer thicknesses. We then used MOKE microscopy to check the magnetization states of the uncompensated SAF sample before and after SOT switching. As shown in supplementary Figure 5a, we first applied a positive saturated magnetic field along the +z direction and remove the magnetic field to initialize the magnetization to ↓↑ state. Next the whole background was subtracted and the grey colour represents ↓↑ magnetization state. After that, a current pulse of +50 mA was applied to the channel and we found that the channel area turned white, representing the ↑↓ state (supplementary Figure 5b). Following this, we applied a −50 mA current pulse to the Hall channel and the white area became grey again, indicating the ↓↑ state (supplementary Figure 5c). The MOKE measurements above further confirm the fully SOT switching in the SAF structure.

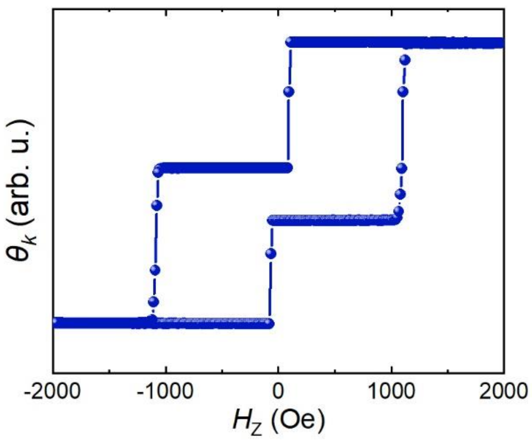

Supplementary Figure 4 | Out-of-plane field (H_z) dependent Kerr signals (θ_k) of the uncompensated SAF sample..

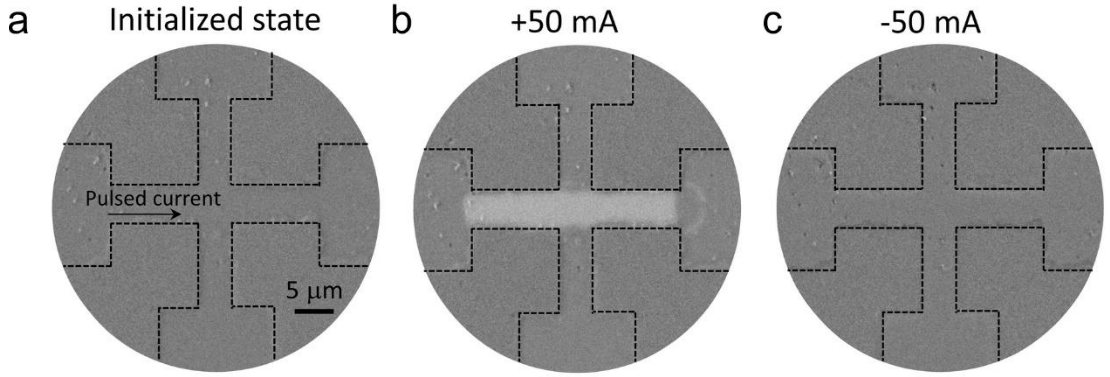

Supplementary Figure 5 | MOKE imaging of the uncompensated SAF specimen during field-free SOT switching. The black dashed lines in the figures outline the Hall bar edges. For process (**a-c**), we first applied a positive saturated magnetic field along the +z direction and remove the magnetic field to initialize the magnetization to ↓↑ state (**a**). Then the whole background was subtracted and the grey colour represents ↓↑ magnetization state. After that, we successively applied +50 mA (**b**) and −50 mA (**c**) to

the Hall channel to check the magnetization evolution. The white-coloured region in (**b**) represents the ↑↓ state.
